# Stress-induced hyperglycemia and mortality of non-diabetic patients with sepsis: a meta-analysis

**DOI:** 10.3389/fendo.2025.1688494

**Published:** 2025-11-11

**Authors:** Jiayang Huang, Junwei Li, Xiaowen Chen, Jiayu Gu

**Affiliations:** 1 Department of Pharmacy, Shenzhen People’s Hospital, The Second Clinical Medical College, Jinan University, Shenzhen, China; 2 The First Affiliated Hospital, Southern University of Science and Technology, Shenzhen, China

**Keywords:** sepsis, stress-induced hyperglycemia, mortality, survival, meta-analysis

## Abstract

Stress-induced hyperglycemia (SIH) is a common metabolic response in critically ill patients, but its prognostic significance in non-diabetic patients with sepsis remains unclear. We conducted a meta-analysis to evaluate the association between SIH and short-term mortality in this population. PubMed, Embase, and Web of Science were searched for relevant observational studies. SIH was defined by study-specific early admission blood glucose cutoffs. Risk ratios (RRs) and 95% confidence intervals (CIs) were pooled using a random-effects model by incorporating the influence of heterogeneity. Thirteen studies encompassing 53,073 non-diabetic septic patients were included. Overall, SIH was associated with significantly increased mortality (RR = 1.75, 95% CI: 1.45–2.11, *p* < 0.001; I² = 72%), with a 95% prediction interval of 1.18–2.61, indicating that the association is expected to persist in most future study populations despite heterogeneity. Subgroup analyses confirmed the association across prospective and retrospective/*post-hoc* studies (RR: 2.01 vs. 1.63, *p* = 0.29), different sepsis severities (RR: 1.60 vs. 2.26, *p* = 0.12), diagnostic criteria (Sepsis-2.0: 1.84 vs. Sepsis-3.0: 1.73, *p* = 0.80), timing of SIH assessment (at admission, within 24 h, or 48 h; *p* = 0.16), and glucose cutoffs (7.8, 11.1, 16.7 mmol/L; *p* = 0.34). Notably, SIH showed a stronger association with ICU/in-hospital mortality (RR = 2.25) compared with 1-month (1.63) or 3–12-month mortality (1.35; *p* = 0.001). Meta-regression showed no significant effect of study characteristics (*p* all > 0.05). In conclusion, SIH defined by early admission blood glucose is significantly associated with increased short-term mortality in non-diabetic septic patients. These findings highlight the importance of early recognition and monitoring of SIH in this high-risk population.

**Systematic review registration:**

https://www.crd.york.ac.uk/PROSPERO/, identifier CRD42024587545.

## Introduction

Sepsis is a life-threatening syndrome caused by dysregulated host responses to infection and remains one of the leading causes of mortality worldwide (1, 2). Despite advances in antimicrobial therapy, supportive care, and intensive care monitoring, short-term mortality in patients with sepsis continues to range from 20% to 40% depending on severity and comorbidities (3). Numerous prognostic models, including the Acute Physiology and Chronic Health Evaluation (APACHE) and Sequential Organ Failure Assessment (SOFA) scores, have been developed to predict mortality risk in septic patients (4, 5). These scores are well-established and widely validated tools for assessing sepsis severity and prognosis, offering recognized advantages in convenience, operability, and accuracy (4, 5). However, APACHE II and SOFA scores primarily capture physiologic and organ dysfunction parameters and do not directly reflect the host’s acute metabolic stress response (6). Therefore, there is a need for novel, simple, and easily measurable biomarkers that can complement existing severity scores by identifying patients with heightened stress responses who are at greater risk of adverse outcomes, thereby improving early risk stratification and guiding timely management strategies.

Stress-induced hyperglycemia (SIH), defined as a transient elevation in blood glucose during acute illness in patients without pre-existing diabetes, is one such candidate factor (7). The pathophysiology of SIH involves increased counter-regulatory hormones, inflammatory cytokine release, and stress-mediated insulin resistance (8). Potential mechanisms linking SIH with adverse outcomes in sepsis include impairment of immune cell function, endothelial injury, oxidative stress, and promotion of a pro-coagulant state, which may aggravate organ dysfunction and increase the risk of death (9, 10). Recent studies have also reported that other metabolic indicators, such as the glucose-potassium ratio (11), the neutrophil-to-prognostic nutritional index ratio (12), and the triglyceride-glucose index (13), are associated with increased mortality in sepsis, further underscoring the clinical relevance of metabolic dysregulation in this condition. The prognostic impact of SIH may differ between patients with and without pre-existing diabetes. In individuals with diabetes, admission hyperglycemia often reflects both chronic dysglycemia and the acute stress response, making it more difficult to isolate the prognostic effect of stress-related hyperglycemia (14). By contrast, in patients without known diabetes, elevated glucose levels at presentation are more likely to represent an acute metabolic response to critical illness. However, previous studies evaluating SIH have frequently included both diabetic and non-diabetic patients. In diabetic patients, the prognostic role of SIH is complicated by baseline hyperglycemia, glycemic variability, and the effects of antidiabetic therapy, which may obscure the true relationship between SIH and mortality. To reduce this potential confounding and to focus on the population in whom SIH is most reflective of stress response, we restricted our analysis to non-diabetic septic patients. Moreover, the evidence regarding the prognostic value of SIH in non-diabetic septic patients remains inconsistent and not universally established (15–27). To address these uncertainties, we performed a systematic review and meta-analysis to investigate the association between SIH, defined by early admission blood glucose levels, and short-term mortality in non-diabetic patients with sepsis.

## Methods

This study followed the PRISMA 2020 (28) and Cochrane Handbook guidelines (29) for conducting systematic reviews and meta-analyses, covering study design, data collection, statistical methods, and interpretation of results. The protocol was also registered in PROSPERO under the ID CRD42024587545.

### Database search

To identify studies pertinent to this meta-analysis, we searched PubMed, Embase, and Web of Science databases using an extensive array of search terms, which involved the combined terms of (1) “stress-induced hyperglycemia” OR “stress induced hyperglycemia” OR “SIH” OR “hyperglycemia”; (2) “sepsis” OR “septic” OR “septicemia”; and (3) “death” OR “deaths” OR “mortality” OR “survival” OR “clinical outcome” OR “prognosis” OR “prospective” OR “retrospective” OR “cohort” OR “follow-up” OR “followed” OR “longitudinal” OR “prospectively” OR “retrospectively”. The search was restricted to studies on human subjects and included only full-length articles published in English in peer-reviewed journals. We also manually checked the references of related original and review articles to find additional relevant studies. The search covered all records from database inception up to June 12, 2025. The search strategy included the term “hyperglycemia” to ensure comprehensive retrieval, as many early studies on SIH in sepsis were indexed under this term. During screening, we carefully evaluated all studies and included only those defining SIH by admission venous glucose levels. The full search strategy for each database is shown in **Supplementary File 1**.

### Study eligible criteria

We applied the PICOS framework to define the inclusion criteria:

P (patients): Adults (≥ 18 y) with sepsis/severe sepsis/septic shock without pre-existing diabetes (ascertained by history/diagnosis codes/antidiabetic medication or HbA1c ≥ 6.5% when reported). For studies reporting outcome in diabetic and non-diabetic patients separately, only data from the non-diabetic patients were analyzed. The diagnostic criteria for sepsis were consistent with the criteria used in the original studies.

I (exposure): SIH defined solely by venous blood glucose using study-defined cutoffs (binary or categorical), measured within 48 hours of patient admission.

C (comparison): Patients normoglycemia or lower admission-glucose category per study definition.

O (outcome): Mortality risk during follow-up, compared between patients with and without SIH.

S (study design): Observational studies with longitudinal follow-up, such as cohort studies (prospective or retrospective), nested case-control studies, and *post-hoc* analyses of clinical trials.

Studies fulfilled either of the following criteria were excluded: (1) studies including pediatric patients, mixed critical-illness cohorts without a separable sepsis subgroup, or patients with pre-existing diabetes or unclear diabetes status when non-diabetic data are not separable; (2) studies not evaluating SIH or reporting only continuous early admission blood glucose without categorical cutoffs; (3) studies not reporting the outcome of mortality; or (4) case reports/series, reviews, meta-analyses, editorials, letters, animal/*in-vitro* studies. If studies had overlapping populations, we included the one with the largest sample size in the meta-analysis.

### Study quality evaluation

Two authors independently performed the literature search, study selection, quality assessment, and data extraction. Disagreements were resolved by discussion with the corresponding author. Study quality was assessed using the Newcastle–Ottawa Scale (NOS) (30), which rates selection, control of confounders, and outcome evaluation. Scores range from 1 to 9, with scores of 8 or higher considered good quality.

### Data collection

The data collected for analysis included the study details (author, year, study country, and design), participant characteristics (number of patients included in each study, diagnosis, diagnostic criteria for sepsis, mean ages of the patients, and the proportion of men), exposure analysis (timing and cutoff values for defining SIH, and numbers of patients with SIH at admission), median follow-up durations, numbers of patients who died during follow-up, and covariates adjusted in the regression models for the analysis of the association between SIH and mortality.

### Statistical analysis

We used risk ratios (RRs) and 95% confidence intervals (CIs) to assess the association between SIH and mortality risk in non-diabetic patients with sepsis. RRs and standard errors were directly extracted or calculated from 95% CIs or p values, then log-transformed to stabilize variance and normalize the data (29). If multiple RRs were reported from different models, we used the one with the most complete adjustment. Heterogeneity was assessed using the Cochrane Q test and I² statistic (31), with a *p* value < 0.10 suggesting significant heterogeneity and I² values of < 25%, 25–75%, and > 75% indicating low, moderate, and high heterogeneity. A random-effects model was used to pool the data, accounting for heterogeneity between studies (29). To further characterize the impact of heterogeneity on the expected range of effects in future studies, we also calculated a 95% prediction interval (PI) for the overall pooled effect size in the random-effects model (29). Sensitivity analyses were done by removing one study at a time to evaluate the robustness of the findings (29). Predefined subgroup analyses were conducted based on study design, severity of sepsis, diagnostic criteria of sepsis, cutoff for the diagnosis of SIH, follow-up durations, and whether the severity scores of sepsis were adjusted. Medians of continuous variables were used to divide subgroups evenly. In addition, a univariate meta-regression analysis was also performed to evaluate the influence of study characteristics on the results, such as sample size, mean ages of the patients, proportions of men, cutoffs for the diagnosis of SIH, and study quality scores (29). Publication bias was assessed using funnel plots and visual inspection for asymmetry, along with Egger’s test (32). All analyses were performed using RevMan (Version 5.3; Cochrane Collaboration, Oxford, UK) and Stata (Version 17.0; Stata Corporation, College Station, TX, USA).

## Results

### Study inclusion

**Figure 1:**
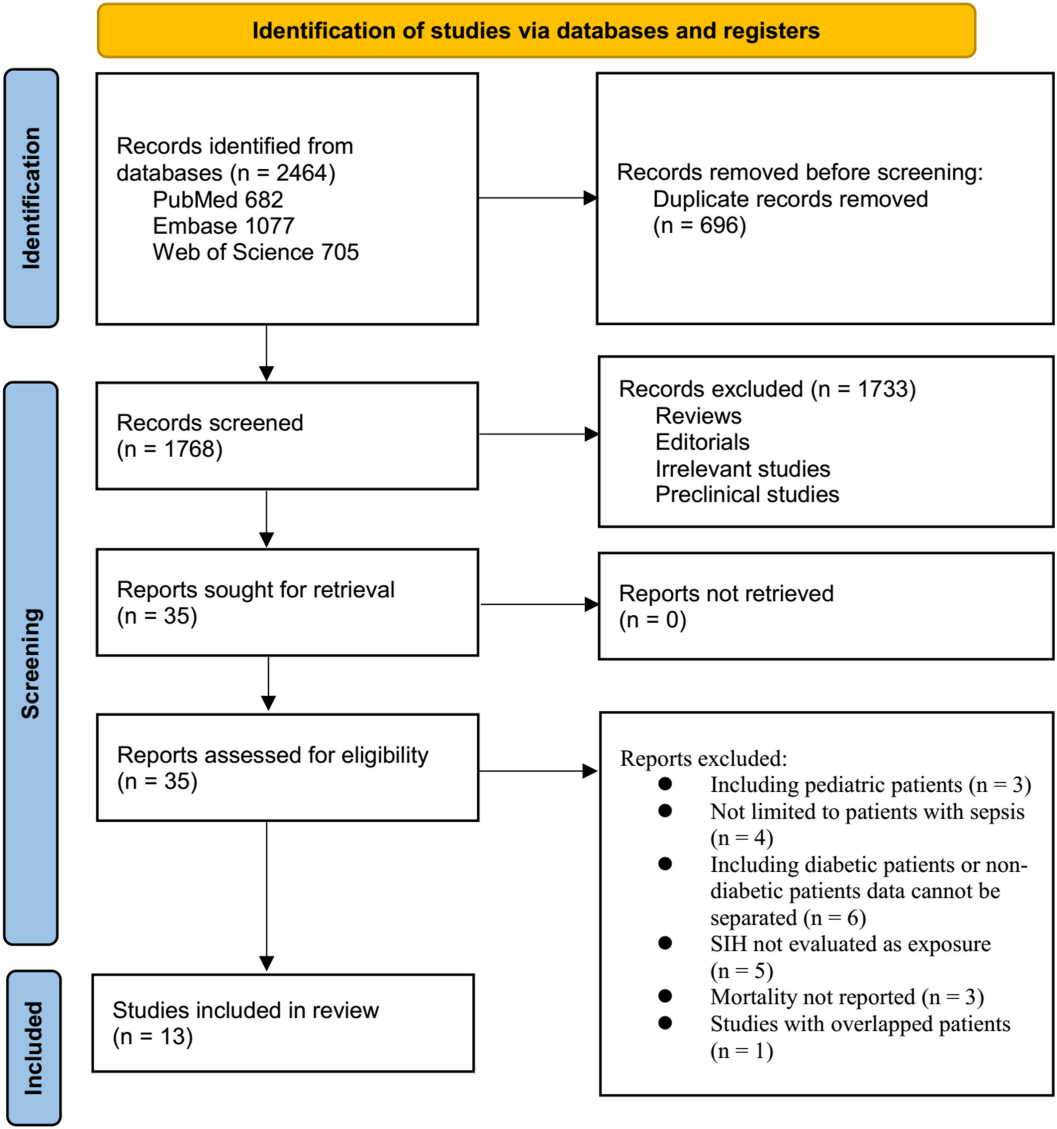
Flowchart of database search and study inclusion.

The study selection process is shown in **Figure 1**. We first identified 2,464 records from the three databases. After removing 696 duplicates, 1,768 articles were screened by title and abstract. Of these, 1,733 were excluded primarily for not meeting the aims of the meta-analysis. The full texts of the remaining 35 articles were reviewed by two independent authors, and 22 were excluded for various reasons (see **Figure 1**). In the end, 13 studies were included in the quantitative analysis (15–27).

### Characteristics of the included studies

**Table 1** summarizes the key characteristics of the 13 studies included in this meta-analysis. The studies were published between 2007 and 2025 and were conducted across diverse regions, including Greece, Thailand, the Netherlands, the United States, Japan, Israel, and China. Five studies were prospective cohort studies (15–17, 19, 22), and the other eight were retrospective cohorts (20, 21, 23–27) or *post-hoc* analysis of a clinical trial (18). Overall, 53,073 non-diabetic patients with sepsis were included in the meta-analysis. Across studies, the mean or median age of participants ranged from 58.7 to 77.0 years, with the proportion of male patients spanning 38.7% to 70.3%. Sepsis was diagnosed according to Sepsis-2.0 criteria in most early studies (15–17, 19–22, 24), Sepsis-3.0 in later studies (23, 25–27), and the PROWESS trial criteria in one study (18). SIH was uniformly defined by admission serum glucose levels, although the specific cutoff values varied: most studies used > 11.1 mmol/L (15, 16, 18–20, 22–27), while others adopted > 7.8 (17) or > 16.7 mmol/L (21). SIH was generally assessed at admission or within the first 24–48 hours. Although most included studies did not report the measurement device, admission venous glucose in clinical practice is typically assessed in the central hospital laboratory using semi- or fully-automated analyzers rather than bedside glucometers. Given that all measurements were obtained in routine clinical settings, the use of standardized laboratory protocols is unlikely to have introduced significant variability or affected the pooled results. Follow-up durations ranged from within ICU or hospitalization to 1 year. Adjustment for confounders differed across studies: earlier smaller cohorts provided unadjusted estimates (15, 17), while larger studies commonly adjusted for age, sex, comorbidities, and severity indices such as SOFA, APACHE, or organ dysfunction variables (16, 18–27).

**Table 1:** Characteristics of the included studies.

Study	Country	Study design	Sample size	Diagnosis	Diagnostic criteria for sepsis	Mean age (years)	Men (%)	Timing of SIH evaluation	Definition of SIH	No. of patients with SIH	Follow-up duration (days)	No. of patients died	Variables adjusted
Leonidou 2007	Greece	PC	35	Severe sepsis without pre-existing DM	Sepsis-2.0	69.3	54.3	Within 24h of admission	> 11.1 mmol/L	16	28	8	None
Leonidou 2008	Greece	PC	200	Severe sepsis without previous existing DM	Sepsis-2.0	70.2	43	Within 24h of admission	> 11.1 mmol/L	47	Within hospitalization	41	Age, SOFA score, infection source, bacteremia
Rattanataweeboon 2009	Thailand	PC	70	Sepsis without pre-existing DM	Sepsis-2.0	64.2	44.3	Within 48 hours of admission	> 7.8 mmol/L	30	30	38	None
Stegenga 2010	11 countries	*Post-hoc* analysis of RCT	642	Severe sepsis without pre-existing DM	Infection + ≥3 SIRS signs + ≥1 organ dysfunction (per PROWESS trial)	59.5	58.3	At admission	> 11.1 mmol/L	93	90	196	Age, APACHE II, SOFA scores, organ failures, and steroid use
Schuetz 2011	USA	PC	5910	Sepsis without pre-existing DM	Sepsis-2.0	59	50	At admission	> 11.1 mmol/L	NR	Within hospitalization	260	Age, sex, sepsis severity (sepsis/severe sepsis/septic shock), disease severity (Mortality in ED Sepsis score, SAPS II)
Green 2013	USA	RC	1236	Sepsis without pre-existing DM	Sepsis-2.0	77	49.7	At admission	> 11.1 mmol/L	115	28	214	Age, CCI, renal dysfunction (BUN >20 mg/dL), respiratory dysfunction (RR >20 or hypoxemia), and cardiovascular dysfunction (SBP <90 mmHg post-fluid).
Kushimoto 2014	Japan	RC	497	Severe sepsis without pre-existing DM	Sepsis-2.0	73	61	Within 24h of admission	> 16.7 mmol/L	16	Within hospitalization	147	Age, sex, DIC score, SOFA score, APACHE II score
van Vught 2016	The Netherlands	PC	767	Sepsis without pre-existing DM	Sepsis-2.0	61.7	60.9	At admission	> 11.1 mmol/L	93	30	76	Age, BMI, modified Charlson comorbidity index, shock on admission, and lactate levels
van Vught 2017	The Netherlands	RC	33407	Sepsis without pre-existing DM	Sepsis-2.0	67	57.3	Within 24h of admission	> 11.1 mmol/L	9159	90	10043	Age, BMI, APACHE III score (modified), admission source, chronic comorbidities (e.g., renal failure, COPD), acute organ failures, and infection type
Chao 2017	Taiwan (China)	RC	2571	Sepsis without pre-existing DM	Sepsis-3.0	65	56.8	At admission	> 11.1 mmol/L	205	Within hospitalization	212	Age, malignancy, hemodialysis, liver disease, infection site, sepsis severity, and glucose variability
Zohar 2021	Israel	RC	1058	Community-onset sepsis without pre-existing DM	Sepsis-3.0	59	38.7	At admission	> 11.1 mmol/L	33	90	130	Age, sex, dementia, clinical syndrome, bacterial growth, comorbidities (e.g., CKD, malignancy), and healthcare exposures
Lu 2022	USA	RC	3699	Sepsis without pre-existing DM	Sepsis-3.0	68	64.4	At admission	> 11.1 mmol/L	132	During ICU stay	595	Age, sex, comorbidities (immunosuppression, liver disease), SOFA/APS III scores, septic shock, hypoglycemia, interventions (MV, RRT, insulin)
Ma 2025	China	RC	2981	Sepsis without pre-existing DM	Sepsis-3.0	58.7	70.3	Within 48 hours of admission	> 11.1 mmol/L	758	365	1674	Age, sex, admission department, CCI, APACHE II, SOFA, creatinine, bilirubin, hemoglobin, hemodynamics, interventions

### Study quality evaluation

**Table 2** presents the quality assessment of the included studies using the NOS. Overall, methodological quality was moderate to high, with total scores ranging from 7 to 9 out of a maximum of 9. Eight studies achieved the score of 8 (16, 18–20, 24, 25, 27) or 9 (22), while the remainder scored 7 (15, 17, 21, 23, 26). Common reasons for slightly lower scores included limited representativeness of the exposed cohort and inadequate follow-up duration. Nevertheless, all studies demonstrated robust ascertainment of exposure, appropriate selection of non-exposed cohorts, reliable outcome assessment. These results support the overall reliability of the mortality data synthesized in this review.

**Table 2:** Study quality evaluation via the Newcastle-Ottawa scale.

Study	Representativeness of the exposed cohort	Selection of the non-exposed cohort	Ascertainment of exposure	Outcome not present at baseline	Control for age	Control for other confounding factors	Assessment of outcome	Enough long follow-up duration	Adequacy of follow-up of cohorts	Total
Leonidou 2007	1	1	1	1	0	0	1	1	1	7
Leonidou 2008	1	1	1	1	1	1	1	0	1	8
Rattanataweeboon 2009	1	1	1	1	0	0	1	1	1	7
Stegenga 2010	0	1	1	1	1	1	1	1	1	8
Schuetz 2011	1	1	1	1	1	1	1	0	1	8
Green 2013	0	1	1	1	1	1	1	1	1	8
Kushimoto 2014	0	1	1	1	1	1	1	0	1	7
van Vught 2016	1	1	1	1	1	1	1	1	1	9
van Vught 2017	0	1	1	1	1	1	1	1	1	8
Chao 2017	0	1	1	1	1	1	1	0	1	7
Zohar 2021	0	1	1	1	1	1	1	1	1	8
Lu 2022	0	1	1	1	1	1	1	0	1	7
Ma 2025	0	1	1	1	1	1	1	1	1	8

### Association between SIH and mortality of non-diabetic patients with sepsis

**Figure 2:**
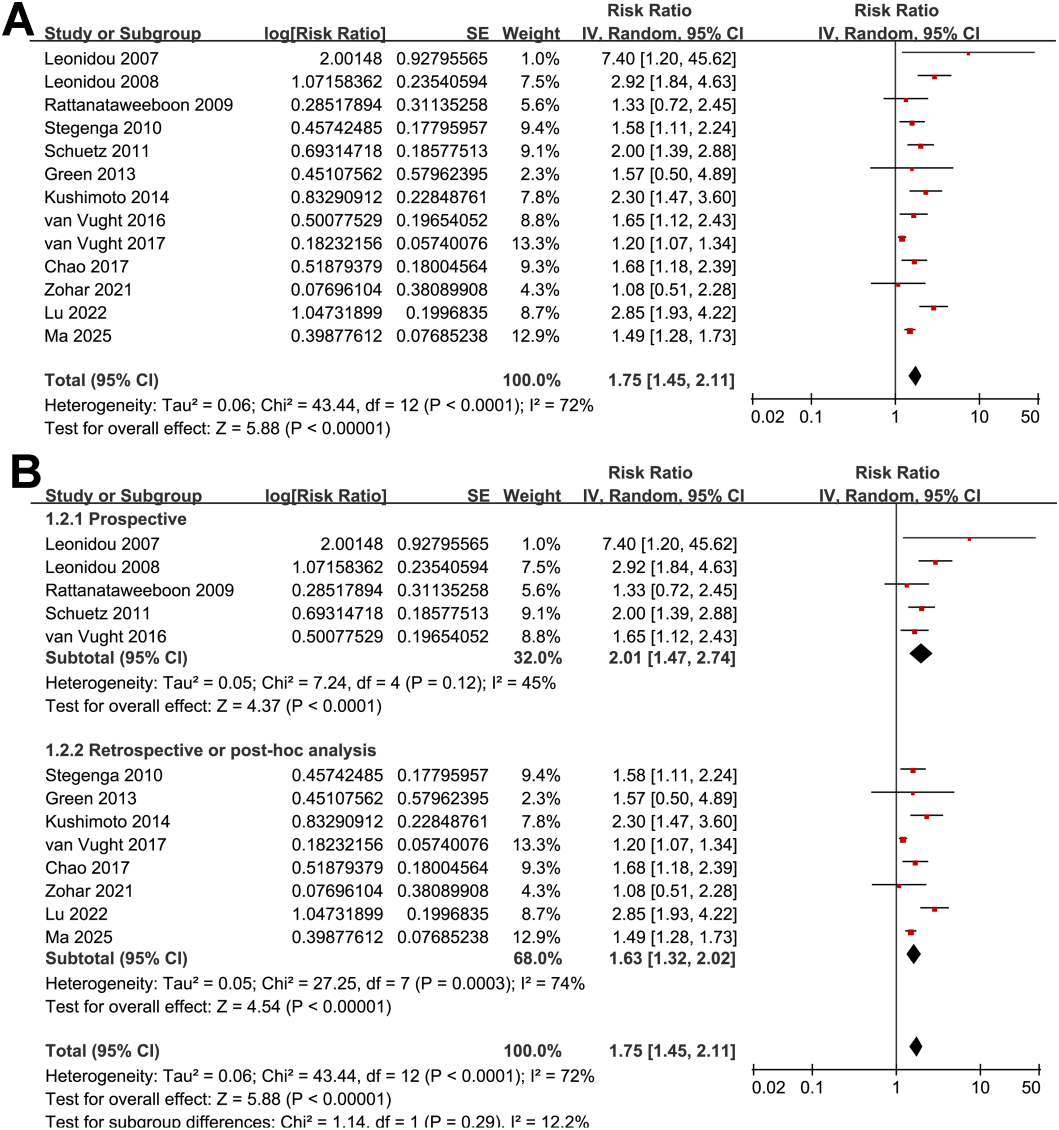
Forest plots for the meta-analysis of the association between SIH and mortality of non-diabetic patients with sepsis; **(A)** overall meta-analysis; and **(B)** subgroup analysis according to study design.

The pooled results of 13 studies (15–27) with a random-effects model showed that overall, SIH was associated with a higher risk of mortality in non-diabetic patients with sepsis (RR: 1.75, 95% CI: 1.45 to 2.11, *p* < 0.001; **Figure 2A**) with moderate heterogeneity (*p* for Cochrane Q test < 0.001; I^2^ = 72%). The 95% PI for the overall pooled estimate was 1.18 to 2.61, indicating that most future studies in comparable clinical settings are expected to demonstrate a positive association between SIH and short-term mortality, although the strength of the association may vary. Sensitivity analyses were performed by removing one dataset at a time, and the results remained stable (RR: 1.66 to 1.85, *p* all < 0.05). Specifically, the sensitivity analysis limited to studies with multivariate analyses only (16, 18–27) showed consistent results (RR: 1.78, 95% CI: 1.47 to 2.17, *p* < 0.001; I^2^ = 75%).

**Figure 3:**
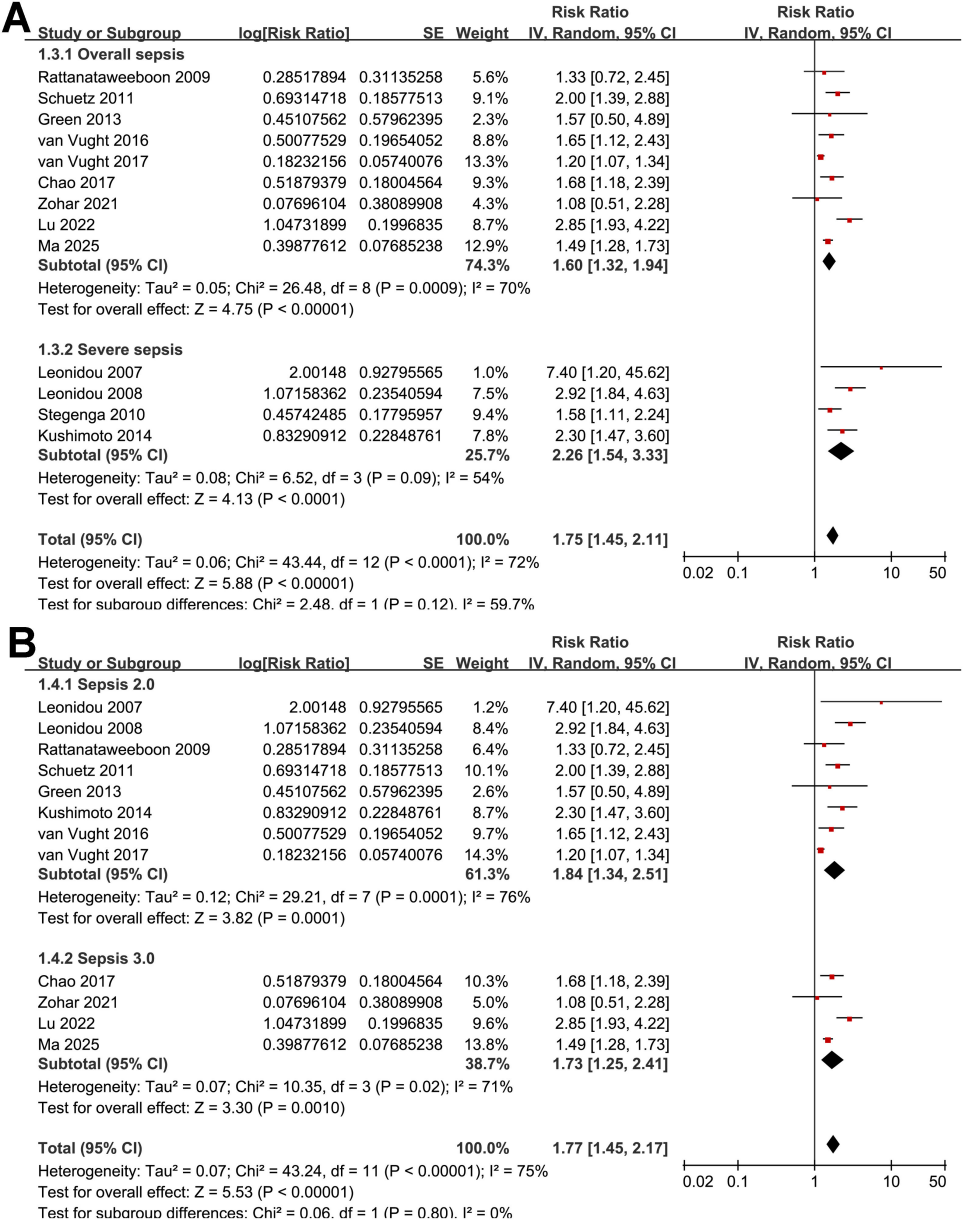
Forest plots for the subgroup analyses of the association between SIH and mortality of non-diabetic patients with sepsis; **(A)** subgroup analysis according to severity of sepsis; and **(B)** subgroup analysis according to diagnostic criteria for sepsis.

**Figure 4:**
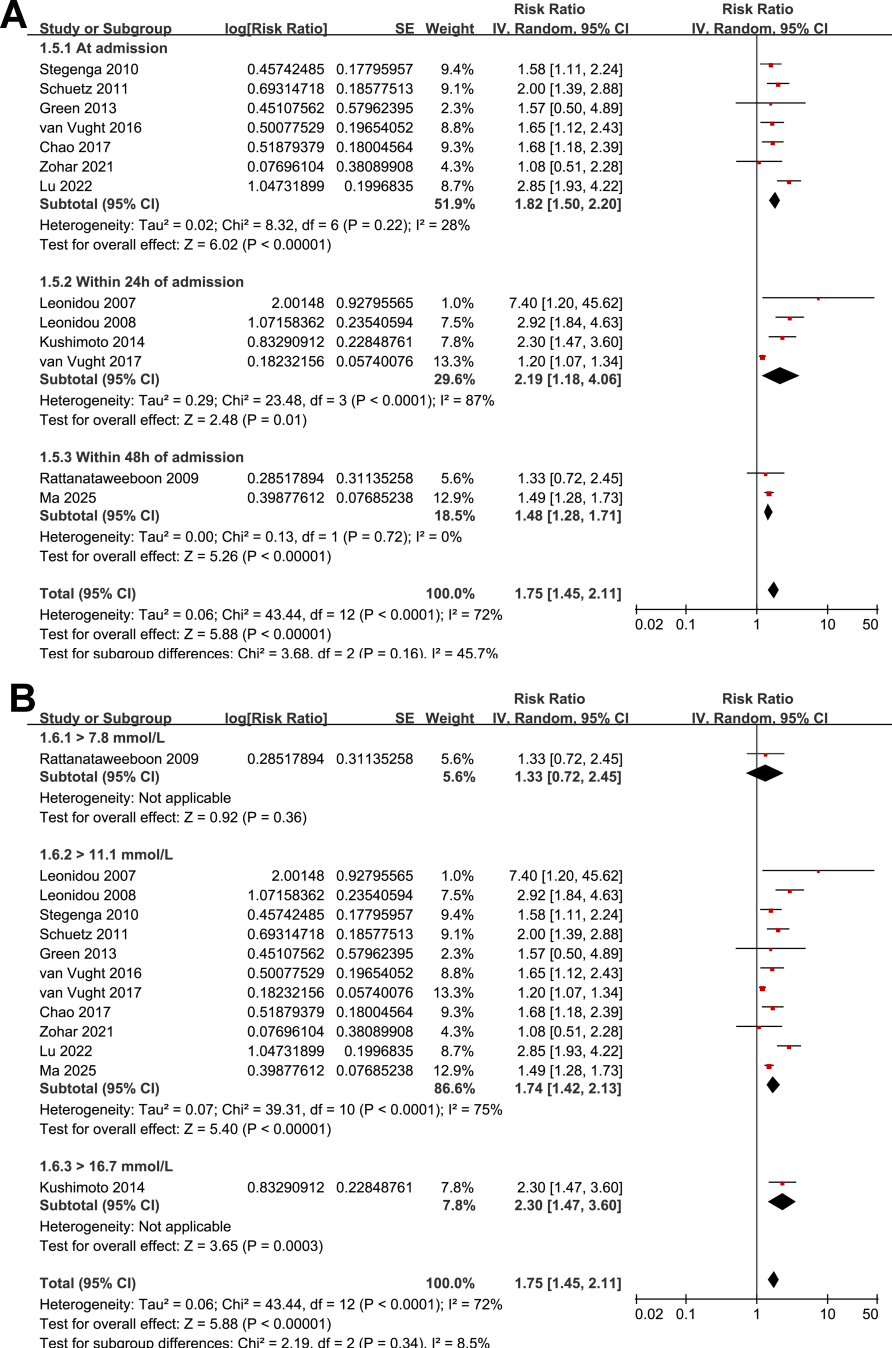
Forest plots for the subgroup analyses of the association between SIH and mortality of non-diabetic patients with sepsis; **(A)** subgroup analysis according to the timing of SIH evaluation; and **(B)** subgroup analysis according to the cutoffs of blood glucose for the diagnosis of SIH.

**Figure 5:**
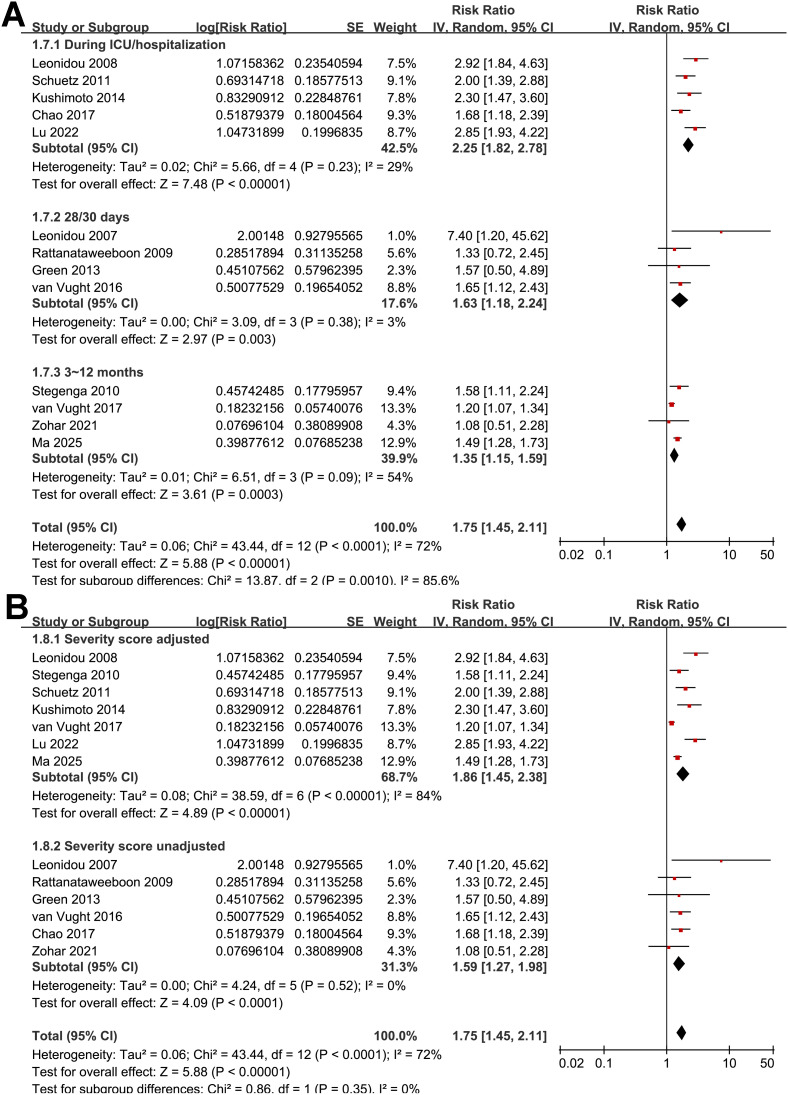
Forest plots for the subgroup analyses of the association between SIH and mortality of non-diabetic patients with sepsis; **(A)** subgroup analysis according to follow-up durations; and **(B)** subgroup analysis by adjustment for severity scores.

Further subgroup analyses indicated that the association between SIH and increased mortality of non-diabetic patients with sepsis was consistent in prospective and retrospective/*post-hoc* studies (RR: 2.01 vs. 1.63, *p* for subgroup difference = 0.29; **Figure 2B**), in patients with overall sepsis and severe sepsis only (RR: 1.60 vs. 2.26, *p* for subgroup difference = 0.12; **Figure 3A**), in studies with sepsis diagnosed with Sepsis-2.0 or -3.0 criteria (RR: 1.84 vs. 1.73, *p* for subgroup difference = 0.80; **Figure 3B**), in SIH evaluated at admission, or within 24 or 48 hours of admission (RR: 1.82, 2.19, vs. 1.48, *p* for subgroup difference = 0.16; **Figure 4A**), and in studies with cutoff for the diagnosis of SIH of 7.8, 11.1, or 16.7 mmol/L (RR: 1.33, 1.74, vs. 2.30, *p* for subgroup difference = 0.34; **Figure 4B**). Interestingly, the association between SIH and mortality appeared strongest for ICU or in-hospital mortality, whereas it was weaker for 1-month and 3–12-month mortality (RR: 2.25, vs. 1.63 and 1.35, *p* for subgroup difference = 0.001; **Figure 5A**), which may partially explain the source of heterogeneity. Similar results were observed for studies with and without the adjustment of severity scores of sepsis (RR: 1.86 vs. 1.59, *p* for subgroup difference = 0.35; **Figure 5B**).

Finally, results of the meta-regression analyses failed to show that differences in study sample size, patient mean age, proportion of men, cutoffs of SIH, or NOS could significantly modify the association between SIH and mortality of non-diabetic patients with sepsis (*p* all > 0.05; **Table 3**). Among them, difference in study sample size may partly explain the between-study heterogeneity (Adjusted R^2^ = 39.5%), although the results were not significant (*p* = 0.09; **Table 3**).

**Table 3:** Results of univariate meta-regression analysis.

Variables	RR for the association between SIH and mortality in non-diabetic patients with sepsis			
	Coefficient	95% CI	P values	Adjusted R^2^
Sample size	-0.0013	-0.0003 to -0.0029	0.09	39.5%
Mean age (years)	0.027	-0.013 to 0.068	0.17	3.8%
Men (%)	0.0003	-0.0249 to 0.0255	0.98	0%
Cutoff of SIH (mmol/L)	0.057	-0.063 to 0.177	0.32	2.7%
NOS	-0.18	-0.52 to 0.16	0.27	6.3%

SIH, stress-induced hyperglycemia; RR, risk ratio; CI, confidence interval; NOS, Newcastle-Ottawa Scale

### Publication bias

**Figure 6:**
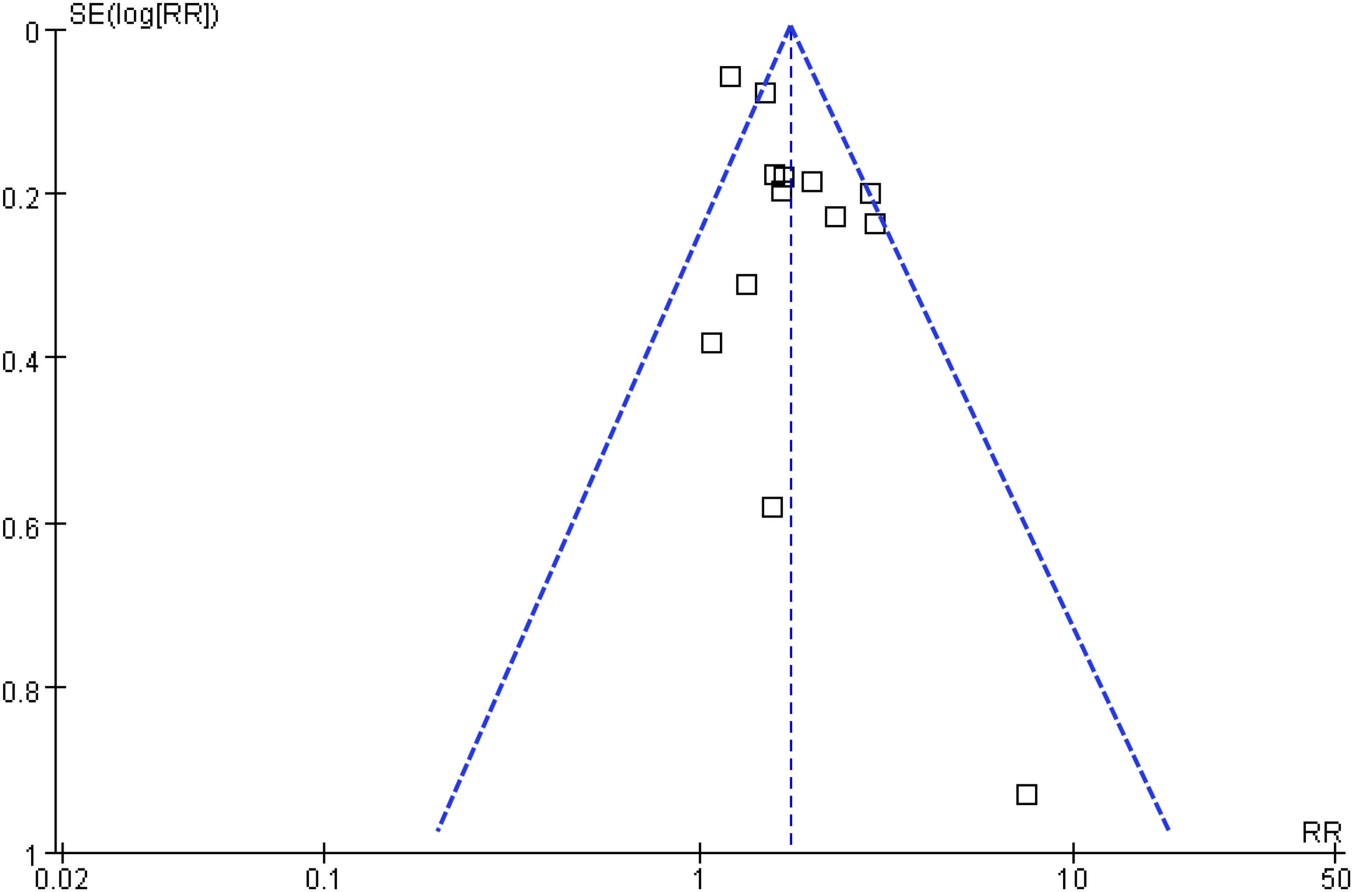
Funnel plots for estimating the potential publication biases underlying the meta-analysis of the association between SIH and mortality of non-diabetic patients with sepsis;.

Funnel plots for the meta-analysis of the association between SIH and mortality risk in non-diabetic patients with sepsis are shown in **Figure 6**. The plots appeared symmetrical, suggesting a low risk of publication bias. Egger’s test also showed no evidence of publication bias (*p* = 0.41).

## Discussion

This meta-analysis demonstrated that SIH, as defined by elevated early admission blood glucose, is associated with a significantly higher risk of short-term mortality in non-diabetic patients with sepsis. The association was consistent across study designs, diagnostic criteria, SIH cutoffs, and timing of glucose evaluation, with sensitivity analyses confirming the robustness of the findings. Importantly, the effect was more pronounced for ICU or in-hospital mortality than for longer-term outcomes, highlighting the immediate impact of acute hyperglycemia during the critical phase of sepsis. These results support SIH as a clinically meaningful prognostic factor that can be easily assessed at admission and may complement established severity scores.

Several pathophysiological mechanisms may explain the link between SIH and mortality in non-diabetic septic patients. Acute stress responses trigger surges in catecholamines, cortisol, and pro-inflammatory cytokines, leading to increased gluconeogenesis, glycogenolysis, and peripheral insulin resistance (8, 33). The resulting hyperglycemia impairs neutrophil function, promotes bacterial proliferation, and disrupts endothelial integrity (34, 35). Hyperglycemia also amplifies oxidative stress, enhances inflammatory cascades, and activates coagulation pathways, thereby exacerbating microvascular injury and multi-organ dysfunction (36). In the absence of pre-existing diabetes, non-diabetic patients lack the adaptive mechanisms that may mitigate glucose fluctuations, potentially rendering them more vulnerable to the harmful effects of acute hyperglycemia (37, 38). The key molecular mechanisms underlying the association between SIH and poor prognosis of patients with critical illnesses warrant further elucidation.

Subgroup analyses showed no significant differences between prospective and retrospective cohorts or across diagnostic criteria, suggesting that the prognostic impact of SIH is independent of study design or sepsis definition. Similarly, the association was observed regardless of whether SIH was measured at admission, within 24 hours, or 48 hours, and across glucose thresholds of 7.8, 11.1, or 16.7 mmol/L. This consistency indicates that the detrimental impact of SIH is not confined to a particular diagnostic cutoff or timing, although more standardized definitions would facilitate comparability across studies. However, the results of subgroup analysis according to the cutoffs of SIH should be interpreted with caution because 11 of the 13 studies used the cutoff of 11.1 mmol/L. Of note, the stronger association with ICU and in-hospital mortality compared with one-month or longer-term outcomes highlights that SIH is primarily an early prognostic marker reflecting acute physiological stress. Finally, the subgroup analysis comparing studies that did and did not adjust for baseline illness severity (e.g., APACHE II, SOFA) revealed a numerically stronger association between SIH and mortality in the adjusted group (RR = 1.86 vs. 1.59), although the between-subgroup difference was not statistically significant. This finding suggests that the adverse prognostic impact of SIH is unlikely to be entirely explained by baseline disease severity, implying a potential pathophysiological contribution of stress-induced hyperglycemia itself—possibly through mechanisms such as immune dysregulation, endothelial injury, and increased inflammatory and pro-coagulant responses. However, given the lack of statistical significance and the observational design of the included studies, this difference should be interpreted cautiously.

Although we performed extensive subgroup and meta-regression analyses, no single study-level factor fully explained the heterogeneity, and differences in sample size accounted for only a modest proportion (adjusted R² = 39.5%). The residual heterogeneity is likely multifactorial and may arise from differences in patient characteristics, such as baseline comorbidities including chronic kidney or liver disease, variations in sepsis severity at presentation, and differences in institutional management protocols for fluid resuscitation, antibiotic stewardship, and glycemic control. In addition, the extent of covariate adjustment for potential confounders varied markedly across studies, ranging from no adjustment in smaller cohorts to extensive adjustment in large databases, potentially influencing effect estimates. To better reflect the practical implications of this heterogeneity, we calculated a 95% PI for the pooled RR, which was 1.18–2.61. This indicates that while the association between SIH and mortality is expected to persist in most future populations, the magnitude of the effect may vary depending on patient characteristics, care protocols, and analytic approaches. These observations underscore the importance of future prospective studies using standardized SIH definitions, uniform reporting of key covariates, and, ideally, individual patient data analyses to further clarify these associations.

This study has several strengths. First, it represents the most comprehensive synthesis to date, including over 53,000 patients across diverse geographic regions and healthcare settings. Second, the analysis was restricted to non-diabetic patients, thereby minimizing confounding from chronic hyperglycemia, underlying metabolic abnormalities, and glucose-lowering treatments. In addition, we performed multiple subgroup, sensitivity, and meta-regression analyses, all of which reinforced the robustness of the primary finding. Nonetheless, some limitations should be acknowledged. Many included studies were retrospective in nature, which may introduce selection bias and limit the completeness of covariate adjustment (39). Another major limitation of the evidence base is the lack of a standardized, universally accepted definition of SIH, as included studies used different glucose cut-off values (7.8, 11.1, or 16.7 mmol/L) and varied in the timing of glucose assessment (at admission, within 24 h, or within 48 h). Although our subgroup analyses did not show significant effect modification by these factors, such inconsistencies may still contribute to residual heterogeneity and limit comparability across studies. Future research should focus on developing and validating consensus definitions for SIH in septic patients to improve the interpretability and applicability of evidence. Similarly, follow-up durations varied across studies, with some reporting in-hospital or ICU mortality and others extending to 1 year, complicating direct comparisons. Although most large cohorts adjusted for important confounders such as severity scores and comorbidities, residual confounding by unmeasured variables cannot be excluded. In particular, most included studies did not report corticosteroid exposure; thus, some non-diabetic patients classified as having SIH may have received corticosteroids, which can induce hyperglycemia and potentially influence prognosis. Moreover, all included studies were observational in design, which precludes establishing causality. While SIH consistently emerged as a significant prognostic marker for short-term mortality, it remains uncertain whether SIH itself directly contributes to adverse outcomes or primarily reflects the underlying severity of sepsis and the host stress response. This distinction is crucial because, without clear evidence of a causal role, SIH cannot yet be considered a modifiable therapeutic target. Future prospective and interventional studies are needed to determine whether targeted glucose management in non-diabetic septic patients can improve outcomes. In addition, the potential heterogeneity in the prognostic impact of SIH across clinically important subgroups—such as elderly patients, pregnant women, or those with underlying liver or kidney dysfunction—could not be explored due to the lack of individual patient data. Future large-scale prospective studies with detailed stratification are warranted to clarify these subgroup-specific associations. Finally, our literature search was restricted to studies published in English and did not include grey literature such as conference proceedings, dissertations, or unpublished data. This approach may introduce language and publication biases, as studies with null or negative findings are less likely to appear in indexed English-language journals. Future systematic reviews on this topic would benefit from incorporating non-English databases and grey literature sources to enhance the comprehensiveness of evidence and reduce the risk of such biases.

From a clinical perspective, these findings emphasize the importance of measuring early admission blood glucose in septic patients without pre-existing diabetes as part of early risk stratification. SIH could serve as a simple, readily available marker to identify high-risk individuals who may benefit from closer monitoring or more aggressive supportive care. However, whether interventions targeting SIH can improve outcomes remains uncertain. Tight glycemic control has been associated with increased risk of hypoglycemia and inconsistent mortality benefits in critically ill populations (40), suggesting that therapeutic strategies must balance the risks of both hyperglycemia and hypoglycemia. Future research should therefore aim to clarify the causal role of SIH in sepsis mortality and to determine whether tailored glucose management strategies in non-diabetic septic patients can modify outcomes. Prospective studies using standardized SIH definitions and stratified analyses by diabetic status are needed, along with interventional trials evaluating glucose control thresholds specific to non-diabetic patients with sepsis.

A key limitation of SIH is that it does not account for chronic glycemia; in populations including patients with undiagnosed diabetes or prediabetes, part of the observed risk may be due to underlying chronic hyperglycemia rather than the acute stress response. To mitigate this limitation, our meta-analysis focused exclusively on non-diabetic septic patients, for whom early admission blood glucose more reliably reflects the stress-induced metabolic response. The stress hyperglycemia ratio (SHR)—calculated as the ratio of admission glucose to HbA1c-estimated chronic glucose—has been proposed to better capture the acute component of hyperglycemia and has been shown to predict adverse outcomes in critically ill and septic patients (41, 42). However, SHR has important shortcomings: it requires HbA1c testing, which is not routinely available in emergency or ICU settings; its reliability can be compromised in critically ill patients due to anemia, transfusions, hemoglobinopathies, or renal dysfunction; and it may delay early bedside risk assessment. In contrast, SIH is measured with routine admission venous glucose, which is universally available, rapid, and cost-effective, making it particularly practical for early risk stratification in non-diabetic septic patients. Future prospective studies should directly compare SIH and SHR to determine whether SHR provides meaningful incremental prognostic value beyond SIH.

## Conclusions

In conclusion, this meta-analysis provides up-to-date evidence that SIH defined by early admission blood glucose is associated with increased short-term mortality in non-diabetic patients with sepsis. The association was consistent across diverse study designs, definitions, and analytic approaches, and was particularly evident for ICU and in-hospital mortality. While these findings highlight the prognostic importance of SIH, limitations of the existing evidence warrant cautious interpretation. Further prospective studies are required to validate these findings and explore the potential benefits of targeted glycemic management in this high-risk population.

## Data Availability

The original contributions presented in the study are included in the article/**Supplementary Materia**l. Further inquiries can be directed to the corresponding author.
