# Supplementary material for: Stress-induced hyperglycemia and mortality of non-diabetic patients with sepsis: a meta-analysis
Source: Front Endocrinol (Lausanne). 2025 Nov 11;16:1688494. doi: 10.3389/fendo.2025.1688494 (PMC12645411; doi:10.3389/fendo.2025.1688494)
Supplement: Supplementary file 1 [file SupplementaryFile1.docx]

**Supplementary File 1** Detailed search strategy for each database

**PubMed**

("stress-induced hyperglycemia"[tiab] OR "stress induced hyperglycemia"[tiab] OR SIH[tiab] OR hyperglyc*[tiab] OR "Hyperglycemia"[Mesh]) AND ("Sepsis"[Mesh] OR sepsis[tiab] OR septic*[tiab] OR "Septicemia"[Mesh] OR septicemia[tiab]) AND ("Mortality"[Mesh] OR "Survival"[Mesh] OR "Prognosis"[Mesh] OR "Treatment Outcome"[Mesh] OR death[tiab] OR deaths[tiab] OR mortality[tiab] OR survival[tiab] OR "clinical outcome*"[tiab] OR prognosis[tiab] OR prospective[tiab] OR retrospectiv*[tiab] OR cohort*[tiab] OR "follow-up"[tiab] OR "follow up"[tiab] OR followed[tiab] OR longitudinal[tiab] OR prospectively[tiab] OR retrospectivey[tiab]) AND humans[MeSH Terms] AND english[lang] AND ("1800/01/01"[Date - Publication] : "2025/06/12"[Date - Publication])

**Embase**

('pregnancy'/exp OR 'pregnant woman'/exp OR pregnancy:ti,ab OR pregnant:ti,ab OR prenatal:ti,ab OR pre-natal:ti,ab) AND ('smoking'/exp OR 'tobacco use'/exp OR smoking:ti,ab OR smoke:ti,ab OR cigarette:ti,ab OR cigarettes:ti,ab OR nicotine:ti,ab OR tobacco:ti,ab) AND ('type 1 diabetes mellitus'/exp OR 'diabetes mellitus':ti,ab OR 'type 1 diabetes':ti,ab OR T1D:ti,ab OR T1DM:ti,ab OR diabetic:ti,ab) AND ('child'/exp OR 'adolescent'/exp OR 'pediatrics'/exp OR child:ti,ab OR children:ti,ab OR adolescent:ti,ab OR adolescents:ti,ab OR pediatric:ti,ab OR paediatric:ti,ab OR offspring:ti,ab OR childhood:ti,ab OR adolescence:ti,ab) AND [humans]/lim AND [article]/lim

**Web of Science**

TS=("pregnancy" OR "pregnant" OR "prenatal" OR "pre-natal") AND TS=("smoking" OR "smoke" OR "cigarette" OR "cigarettes" OR "nicotine" OR "tobacco") AND TS=("type 1 diabetes" OR "diabetes" OR "diabetic" OR "T1D" OR "T1DM") AND TS=("child" OR "children" OR "adolescent" OR "adolescents" OR "pediatric" OR "paediatric" OR "offspring" OR "childhood" OR "adolescence") AND DT=(Article) AND LA=(English)
